# Central Ohio Dental Association

**Published:** 1866-05

**Authors:** 


### CENTRAL OHIO DENTAL ASSOCIATION.

The regular annual meeting of this body, was held on the 8th of May, at Mt. Vernon, Ohio. This is a live society—its members are all earnest, industrious workers for the good of the profession. Several prominent subjects of general interest were taken up and discussed, and such action taken as will tend to push them on to the desired ultimatum. And not least among these was the procurement of a law, that shall protect the people against dental charlatanism, and stimulate the members of the profession to make greater advances in their ability to do good. The society increased its membership largely. Its operations must result largely for good, for it embraces a field that has been almost entirely shut out from the advantages of association, chiefly because of locality. This society will revolutionize the profession in the central and south-eastern part of the State, and that very soon too.

The semi-annual meeting is held in Zanesville, on the second Tuesday of November next, when there will, we doubt not, be a large meeting.

